# Genome-wide meta-analysis identifies multiple novel loci associated with serum uric acid levels in Japanese individuals

**DOI:** 10.1038/s42003-019-0339-0

**Published:** 2019-04-08

**Authors:** Masahiro Nakatochi, Masahiro Kanai, Akiyoshi Nakayama, Asahi Hishida, Yusuke Kawamura, Sahoko Ichihara, Masato Akiyama, Hiroaki Ikezaki, Norihiro Furusyo, Seiko Shimizu, Ken Yamamoto, Makoto Hirata, Rieko Okada, Sayo Kawai, Makoto Kawaguchi, Yuichiro Nishida, Chisato Shimanoe, Rie Ibusuki, Toshiro Takezaki, Mayuko Nakajima, Mikiya Takao, Etsuko Ozaki, Daisuke Matsui, Takeshi Nishiyama, Sadao Suzuki, Naoyuki Takashima, Yoshikuni Kita, Kaori Endoh, Kiyonori Kuriki, Hirokazu Uemura, Kokichi Arisawa, Isao Oze, Keitaro Matsuo, Yohko Nakamura, Haruo Mikami, Takashi Tamura, Hiroshi Nakashima, Takahiro Nakamura, Norihiro Kato, Koichi Matsuda, Yoshinori Murakami, Tatsuaki Matsubara, Mariko Naito, Michiaki Kubo, Yoichiro Kamatani, Nariyoshi Shinomiya, Mitsuhiro Yokota, Kenji Wakai, Yukinori Okada, Hirotaka Matsuo

**Affiliations:** 1 Data Science Division, Data Coordinating Center, Department of Advanced Medicine, Nagoya University Hospital, Nagoya, 466-8560 Japan; 2 Laboratory for Statistical Analysis, RIKEN Center for Integrative Medical Sciences, Yokohama, 230-0045 Japan; 3 Department of Statistical Genetics, Osaka University Graduate School of Medicine, Suita, 565-0871 Japan; 4 Department of Biomedical Informatics, Harvard Medical School, Boston, MA 02115 USA; 5 Department of Integrative Physiology and Bio-Nano Medicine, National Defense Medical College, Tokorozawa, 359-8513 Japan; 6 Medical Squadron, Air Base Group, Western Aircraft Control and Warning Wing, Japan Air Self-Defense Force, Kasuga, 816-0804 Japan; 7 Department of Preventive Medicine, Nagoya University Graduate School of Medicine, Nagoya, 466-8550 Japan; 8 Department of General Medicine, National Defense Medical College, Tokorozawa, 359-8513 Japan; 9 Department of Environmental and Preventive Medicine, Jichi Medical University School of Medicine, Shimotsuke, 329-0498 Japan; 10 Department of Ophthalmology, Graduate School of Medical Sciences, Kyushu University, Fukuoka, 812-8582 Japan; 11 Department of General Internal Medicine, Kyushu University Hospital, Fukuoka, 812-8582 Japan; 12 Department of Medical Biochemistry, Kurume University School of Medicine, Kurume, 830-0011 Japan; 13 Laboratory of Genome Technology, Institute of Medical Science, The University of Tokyo, Tokyo, 108-8639 Japan; 14 Department of Urology, National Defense Medical College, Tokorozawa, 359-8513 Japan; 15 Department of Preventive Medicine, Faculty of Medicine, Saga University, Saga, 849-8501 Japan; 16 International Island and Community Medicine, Kagoshima University Graduate School of Medical and Dental Sciences, Kagoshima, 890-8544 Japan; 17 Department of Surgery, National Defense Medical College, Tokorozawa, 359-8513 Japan; 18 Department of Epidemiology for Community Health and Medicine, Kyoto Prefectural University of Medicine, Kyoto, 602-8566 Japan; 19 Department of Public Health, Nagoya City University Graduate School of Medical Sciences, Nagoya, 467-8602 Japan; 20 Department of Health Science, Shiga University of Medical Science, Otsu, 520-2192 Japan; 21 Department of Nursing, Tsuruga City College of Nursing, Fukui, 914-8501 Japan; 22 Laboratory of Public Health, Division of Nutritional Sciences, School of Food and Nutritional Sciences, University of Shizuoka, Shizuoka, 422-8526 Japan; 23 Department of Preventive Medicine, Institute of Biomedical Sciences, Tokushima University Graduate School, Tokushima, 770-8503 Japan; 24 Division of Cancer Epidemiology and Prevention, Aichi Cancer Center Research Institute, Nagoya, 464-8681 Japan; 25 Department of Epidemiology, Nagoya University Graduate School of Medicine, Nagoya, 466-8550 Japan; 26 Cancer Prevention Center, Chiba Cancer Center Research Institute, Chiba, 260-8717 Japan; 27 Department of Preventive Medicine and Public Health, National Defense Medical College, Tokorozawa, 359-8513 Japan; 28 Laboratory for Mathematics, National Defense Medical College, Tokorozawa, 359-8513 Japan; 29 Department of Gene Diagnostics and Therapeutics, Research Institute, National Center for Global Health and Medicine, Tokyo, 162-8655 Japan; 30 Department of Computational Biology and Medical Sciences, Graduate School of Frontier Sciences, The University of Tokyo, Tokyo, 108-8639 Japan; 31 Division of Molecular Pathology, Institute of Medical Science, The University of Tokyo, Tokyo, 108-8639 Japan; 32 Department of Internal Medicine, School of Dentistry, Aichi Gakuin University, Nagoya, 464-8651 Japan; 33 Department of Oral Epidemiology, Hiroshima University Graduate School of Biomedical & Health Sciences, Hiroshima, 734-8553 Japan; 34 RIKEN Center for Integrative Medical Sciences, Yokohama, 230-0045 Japan; 35 Center for Genomic Medicine, Kyoto University Graduate School of Medicine, Kyoto, 606-8507 Japan; 36 Department of Genome Science, School of Dentistry, Aichi Gakuin University, Nagoya, 464-8651 Japan; 37 Laboratory of Statistical Immunology, Immunology Frontier Research Center (WPI-IFReC), Osaka University, Suita, 565-0871 Japan

## Abstract

Gout is a common arthritis caused by elevated serum uric acid (SUA) levels. Here we investigated loci influencing SUA in a genome-wide meta-analysis with 121,745 Japanese subjects. We identified 8948 variants at 36 genomic loci (*P*<5 × 10^–8^) including eight novel loci. Of these, missense variants of *SESN2* and *PNPLA3* were predicted to be damaging to the function of these proteins; another five loci—*TMEM18*, *TM4SF4*, *MXD3-LMAN2*, *PSORS1C1-PSORS1C2*, and *HNF4A*—are related to cell metabolism, proliferation, or oxidative stress; and the remaining locus, *LINC01578*, is unknown. We also identified 132 correlated genes whose expression levels are associated with SUA-increasing alleles. These genes are enriched for the UniProt transport term, suggesting the importance of transport-related genes in SUA regulation. Furthermore, trans-ethnic meta-analysis across our own meta-analysis and the Global Urate Genetics Consortium has revealed 15 more novel loci associated with SUA. Our findings provide insight into the pathogenesis, treatment, and prevention of hyperuricemia/gout.

## Abstract (web summary)

Masahiro Nakatochi et al. report a genome-wide meta-analysis of serum uric acid levels in the Japanese population. They identify 36 loci, including eight previously unreported loci, suggesting key cellular processes that contribute to elevated serum uric acid levels, which can lead to gout.

## Introduction

Serum uric acid (SUA) is reported to have an antioxidative effect^1,2^, whereas elevated SUA, or hyperuricemia, results in crystal deposition and causes gout^3^. Gout is a common disease characterized by noninfectious acute arthritis. Both gout and hyperuricemia can result from an unhealthful lifestyle^4–6^, but recent genetic studies, including genome-wide association studies (GWASs), have also revealed a genetic contribution to the development of these conditions, with this contribution being larger than that for other common diseases^7–12^. Moreover, epidemiologic studies have revealed their relationship among other diseases such as cardiovascular diseases^13,14^, indicating the importance of elucidation of the pathophysiology of these conditions. To date, several GWASs of SUA have been performed with Caucasian populations^15–24^ as well as Asian populations including Japanese subjects^25,26^. Although there are genetic differences between Caucasian and Asian populations, they have many shared associated genes^3,27^ that exert major effects, such as *ABCG2*, *SLC2A9*, and *SLC22A12*, all of which are well-known representative urate transporters in humans and which are important as therapeutic target molecules for gout and hyperuricemia. Therefore, identifying new loci may not only help elucidate the pathophysiology of these diseases, but may also reveal their target molecules, taking into account the fact that these diseases have a broader genetic basis than other common diseases as described above. Furthermore, the gene expression patterns to which the identified loci contribute should enable us to estimate effective pathways for drug delivery. In the present study, we have investigated the genetic loci that influence SUA with more than 120,000 Japanese individuals in a genome-wide meta-analysis and have compared our findings with those of previous GWASs^24,28^. We identified 36 loci for SUA, including eight previously unreported loci, that suggest key cellular processes which contribute to elevated serum uric acid levels, followed by the identification of 15 more loci by trans-ethnic meta-analysis.

## Results

### Genome-wide meta-analysis

**Fig. 1:**
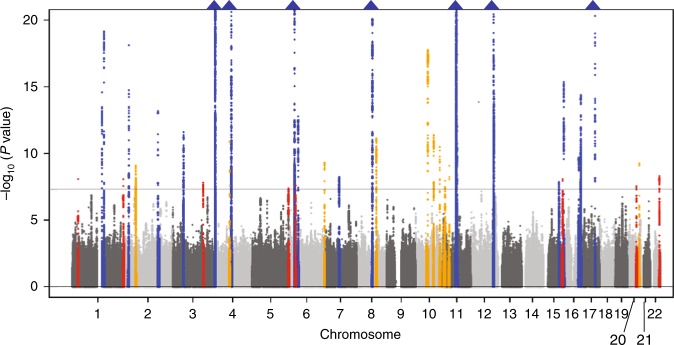
Manhattan plot for the meta-analysis of SUA. The horizontal line represents the genome-wide significance level (*α* = 5 × 10^−8^). Eighteen loci shown in orange were also recently identified by BBJ as being associated with SUA, 10 loci in blue were also identified by other studies and those in red indicate eight novel loci identified in the present study. Blue triangles represent loci containing SNPs with *P* values of <1 × 10^−20^. SUA serum uric acid, BBJ BioBank Japan

We performed a genome-wide meta-analysis based on three Japanese cohorts including those of the Japan Multi-institutional Collaborative Cohort (J-MICC) Study^29,30^, the Kita-Nagoya Genomic Epidemiology (KING) Study^31,32^, and the BioBank Japan (BBJ) Project^33,34^. Detailed information regarding the baseline characteristics of the study subjects, genotyping arrays, and imputation is summarized in Supplementary Tables 1 and 2. We performed a genome-wide meta-analysis for SUA with data sets encompassing 121,745 Japanese subjects. Intercepts of linkage disequilibrium (LD) score regression and the genomic control lambda for each study are shown in Supplementary Table 2. The intercepts of LD score regression and the genomic control lambda for our meta-analysis were 1.043 and 1.165, respectively. Genomic control adjustment was not applied for genomic control at the level of each study because intercepts of LD score regression did not show inflation of test statistics. The quantile–quantile (Q–Q) plot for *P* values is shown in Supplementary Fig. 1. The results of the meta-analysis identified 8948 variants at 36 genetic loci with a *P* value of <5 × 10^–8^ for SUA (Fig. 1). Among these 36 genetic loci, 8 were not previously reported, 10 were recently identified in a GWAS for SUA in Japanese performed by BBJ^28^, and 18 were previously identified by other GWASs for SUA^15–17,19,20,24–26^. The eight novel loci were the following: rs74896528 of *SESN2*, rs10188118 of *LOC105373352* - *TMEM18*, rs6774054 of *TM4SF4*, rs11952102 of *MXD3*-*LMAN2*, rs16898823 of *PSORS1C1*-*PSORS1C2*, rs8024067 of *LINC01578*, rs6031598 of *HNF4A*, and rs2281293 of *PNPLA3*.

**Fig. 2:**
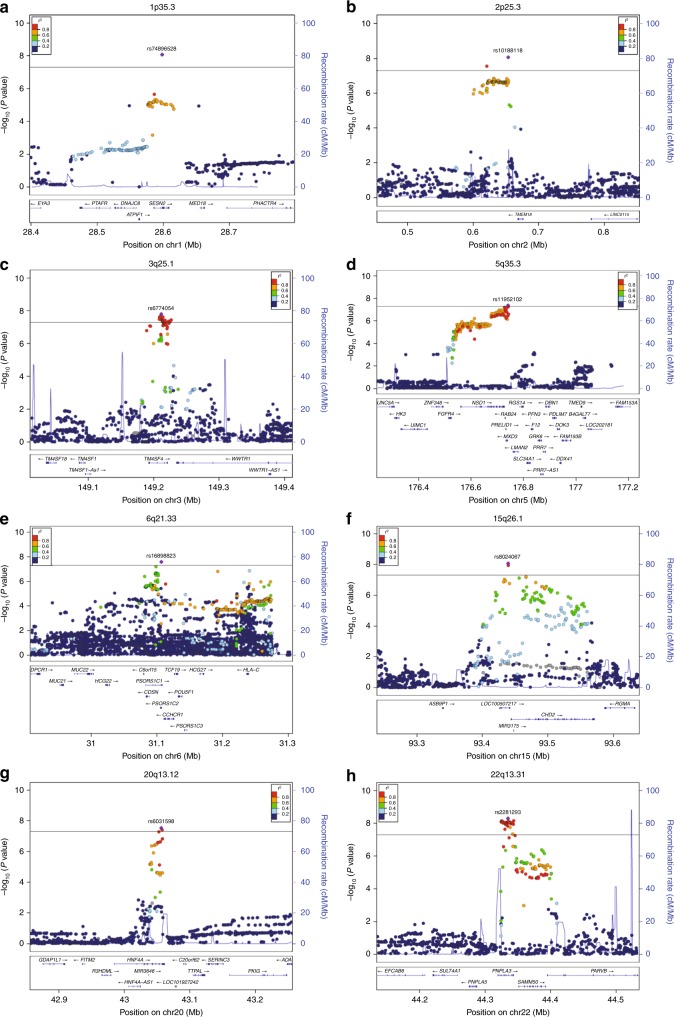
Regional association plots for the eight novel loci identified in the meta-analysis of SUA. The vertical axis represents –log_10_(*P* value) for assessment of the association of each SNP with SUA. Panels **a**–**h** present plots for chromosome (chr) 1p35.3, 2p25.3, 3q25.1, 5q35.3, 6p21.33, 15q26.1, 20q13.12, or 22q13.31, respectively. Colors indicate LD (*r*^2^) between each sentinel SNP and neighboring SNPs based on JPT of 1000 Genomes phase 3. SUA serum uric acid

Sentinel single-nucleotide polymorphisms (SNPs) with the lowest *P* values for SUA at each of the 36 loci are shown in Table 1. Association results of each study are shown in Supplementary Data 1. We determined the effect allele frequencies (EAFs) of these sentinel SNPs for each population in 1000 Genomes phase 3 (Supplementary Data 2). The EAFs indicated that rs74896528 of *SESN2* at chromosome 1p35.3 is an East Asian–specific SNP. Regional association plots for the eight loci newly identified in the present study are shown in Fig. 2. The BBJ data recently revealed that SNPs located at 27 loci showed genome-wide significant associations with SUA including 10 novel loci (Table 1)^28^. About the 27 reported SNPs, we compared the results in our meta-analysis with the recent results by BBJ^28^ (Supplementary Data 3), with regional association plots for the 10 loci also identified in the present study being shown in Supplementary Figure 2. The results for these 27 SNPs, identified in our meta-analysis, revealed a higher level of significance for the association with SUA in our meta-analysis than in the BBJ study. A European GWAS for SUA was previously performed by the Global Urate Genetics Consortium (GUGC)^24^. We examined the publicly available data provided by the GUGC-based study for the sentinel SNPs or SNPs showing high LD (*r*^2^ of ≥0.8 in JPT of 1000 Genomes phase 3) with the sentinel SNPs at the eight novel loci identified in the present study. Three of these loci, including 5q35.3, 20q13.12, and 22q13.31, were significantly associated with SUA in the GUGC-based GWAS, with the same direction of effect size as in our study (Supplementary Table 3). The 2p25.3 locus was nominally significantly associated with SUA. Although the 3q25.1 locus was not significantly associated with SUA in the GUGC-based study, it was nominally significantly associated with gout in the same study.

**Table 1:** Sentinel SNPs associated with SUA in Japanese as identified in the meta-analysis

SNP	Locus	Chr	Position	Gene	Alleles		EAF	Beta^a^ ± SE	*P* value	*I* ^2^
					Effect	Noneffect				
*Novel loci*										
rs74896528	1p35.3	1	28598287	*SESN2*	T	C	0.057	−0.057 ± 0.010	8.42 × 10^−9^	0
rs10188118	2p25.3	2	653623	*LOC105373352, TMEM18*	C	G	0.864	0.035 ± 0.006	8.60 × 10^−9^	48.7
rs6774054	3q25.1	3	149211699	*TM4SF4*	A	G	0.337	0.024 ± 0.004	1.58 × 10^−8^	0
rs11952102	5q35.3	5	176740704	*MXD3, LMAN2*	A	G	0.448	0.022 ± 0.004	4.24 × 10^−8^	0
rs16898823	6p21.33	6	31106606	*PSORS1C1, PSORS1C2*	A	T	0.900	0.037 ± 0.007	2.55 × 10^−8^	0
rs8024067	15q26.1	15	93439224	*LINC01578*	T	G	0.158	−0.034 ± 0.006	8.41 × 10^−9^	40.9
rs6031598	20q13.12	20	43056149	*HNF4A*	T	G	0.378	−0.023 ± 0.004	2.90 × 10^−8^	27.5
rs2281293	22q13.31	22	44334842	*PNPLA3*	T	C	0.559	0.024 ± 0.004	4.99 × 10^−9^	0
*Loci also identified by BBJ* ^28^										
rs811372	2p15	2	61429568	*USP34*	T	C	0.367	0.026 ± 0.004	7.97 × 10^−10^	4
rs10857147	4q21.21	4	81181072	*PRDM8, FGF5*	A	T	0.699	0.032 ± 0.005	1.31 × 10^−11^	17.2
rs13230625	7p22.3	7	1286244	*UNCX, MICALL2*	A	G	0.318	0.027 ± 0.004	4.82 × 10^−10^	0
rs7835379	8q22.1	8	95975080	*TP53INP1, NDUFAF6*	A	G	0.755	0.032 ± 0.005	7.41 × 10^−12^	46.6
rs9416703	10q21.1	10	60283008	*BICC1*	A	C	0.525	−0.036 ± 0.004	1.70 × 10^−18^	0
rs11202346	10q23.2	10	88908912	*FAM35A*	T	G	0.225	0.035 ± 0.005	4.12 × 10^−12^	0
rs1886603	10q26.11	10	119482303	*EMX2, RAB11FIP2*	A	G	0.374	0.027 ± 0.004	3.22 × 10^−11^	0
rs2220970	11p15.4	11	9857749	*SBF2*	A	G	0.342	0.024 ± 0.004	1.12 × 10^−8^	0
rs963837	11p14.1	11	30749090	*MPPED2, DCDC1*	T	C	0.656	0.028 ± 0.005	8.41 × 10^−10^	5.6
rs6026578	20q13.32	20	57463472	*LOC101927932*	C	G	0.278	0.028 ± 0.005	5.48 × 10^−10^	0
*Loci also identified by other studies*										
rs1797052	1q21.1	1	145727683	*PDZK1*	T	C	0.185	0.041 ± 0.005	2.57 × 10^−15^	0
rs4072037	1q22	1	155162067	*MUC1*	T	C	0.828	−0.048 ± 0.005	6.93 × 10^−20^	62.7
rs1260326	2p23.3	2	27730940	*GCKR*	T	C	0.559	0.036 ± 0.004	7.56 × 10^−19^	0
rs16856823	2q31.1	2	170200452	*LRP2*	A	T	0.808	−0.039 ± 0.005	6.61 × 10^−14^	0
rs6445559	3p21.1	3	53099466	*SFMBT1, RFT1*	A	G	0.561	0.029 ± 0.004	2.53 × 10^−12^	2.6
rs7679724	4p16.1	4	9985376	*SLC2A9*	T	G	0.586	0.130 ± 0.004	1.67 × 10^−224^	81.6
rs4148155	4q22.1	4	89054667	*ABCG2*	A	G	0.705	−0.115 ± 0.004	2.05 × 10^−149^	0
rs2762353	6p22.2	6	25794431	*SLC17A1*	A	G	0.160	−0.054 ± 0.005	8.68 × 10^−24^	11.5
rs9394948	6p21.1	6	43334755	*ZNF318*	A	C	0.341	0.032 ± 0.004	1.65 × 10^−13^	9
rs17145750	7q11.23	7	73026378	*MLXIPL*	T	C	0.102	−0.038 ± 0.007	5.85 × 10^−9^	0
rs1828911	8q21.11	8	76462547	*HNF4G*	T	C	0.575	−0.038 ± 0.004	8.08 × 10^−21^	0
rs57633992	11q13.1	11	64424967	*NRXN2*	A	C	0.054	−0.668 ± 0.010	<1 × 10^−300^	68.8
rs79105258	12q24.12	12	111718231	*CUX2*	A	C	0.254	−0.078 ± 0.005	1.91 × 10^−56^	0
rs73436803	15q24.2	15	75619201	*GOLGA6D, COMMD4*	T	C	0.099	−0.043 ± 0.008	1.38 × 10^−8^	0
rs4966024	15q26.3	15	99295570	*IGF1R*	A	G	0.486	−0.033 ± 0.004	4.43 × 10^−16^	0
rs244423	16q22.1	16	69610002	*NFAT5*	A	G	0.156	0.035 ± 0.006	2.00 × 10^−10^	40.5
rs73575095	16q23.2	16	79750332	*MAF, MAFTRR*	T	C	0.719	0.035 ± 0.005	4.03 × 10^−15^	0
rs9895661	17q23.2	17	59456589	*BCAS3*	T	C	0.475	0.044 ± 0.005	9.20 × 10^−23^	0

*Chr* chromosome, *SUA* serum uric acid

^a^The beta value represents change in *z*-score per effect allele copy for the SNP

### Functional annotations for novel loci

We searched for SNPs at the newly identified loci associated with SUA that were associated with gene expression level or amino acid substitution of protein and that were in high LD (*r*^2^ of ≥0.8 in JPT of 1000 Genomes phase 3) with sentinel SNPs and had a *P* value of <1 × 10^–6^ for SUA in our meta-analysis. We identified two nonsynonymous SNPs of *SESN* at the 1p35.3 locus and *PNPLA3* at the 22q13.31 locus (Supplementary Table 4), and we found that six of the eight novel loci harbor variants with expression quantitative trait loci (eQTLs) for at least one tissue in the Genotype-Tissue Expression (GTEx) database^35^ (Supplementary Data 4). The two nonsynonymous SNPs, rs738409 (I148M) of *PNPLA3*, and rs74896528 (P87S) of *SESN2*, were predicted by SIFT, PolyPhen2 HVAR, and PolyPhen2 HDIV to be damaging or probably damaging.

### Gene set enrichment analysis of SUA-associated loci

We searched for genes whose expression level was associated with SUA-associated SNPs in at least one tissue in the GTEx database. We found that 24 of the 36 loci identified in the present study harbor variants with eQTLs in at least one tissue in the GTEx database. We also identified 71 positively correlated genes whose expression level is increased by SUA-increasing alleles and 76 negatively correlated genes whose expression level is decreased by SUA-increasing alleles (Supplementary Data 5). Functional analysis of the sets of positively correlated genes and negatively correlated genes were performed with the Database for Annotation, Visualization, and Integrated Discovery (DAVID)^36^. For the positively correlated genes, the terms “Williams-Beuren syndrome”, “sodium”, “transport”, “sodium transport”, and “alternative splicing” were enriched (Supplementary Table 5). For the negatively correlated genes, the term “Williams–Beuren syndrome” was enriched.

### Comparison between Japanese and European GWASs for SUA

SNPs located at 28 loci were recently found to show genome-wide significant associations with SUA based on data from individuals of European ancestry in the GUGC^24^. We examined the results obtained for these SNPs in our meta-analysis (Supplementary Data 6). Twenty-one of these 25 SNPs showed nominal or genome-wide significant associations with SUA in our meta-analysis, with the same direction of effect size in both studies.

We compared the SNP-based heritability (*h*^*2*^) of SUA in our Japanese meta-analysis and the GUGC-based study^24^. The heritability estimates were calculated from summary statistics of 1,447,573 SNPs, which were assessed in both studies and have MAF ≥1% in both studies. The *h*^*2*^ (standard error (SE)) estimates were 14.0 % (4.3%) for our Japanese study and 14.4% (3.9%) for the European study. Furthermore, we calculated the genetic correlation between Japanese and European studies employing the same data sets. The genetic correlation *ρ*_ge_ (SE) was analyzed (0.591 (0.294), *P* value = 0.164), and was not significantly less than 1.

### Trans-ethnic meta-analysis with the use of GUGC-based study

**Fig. 3:**
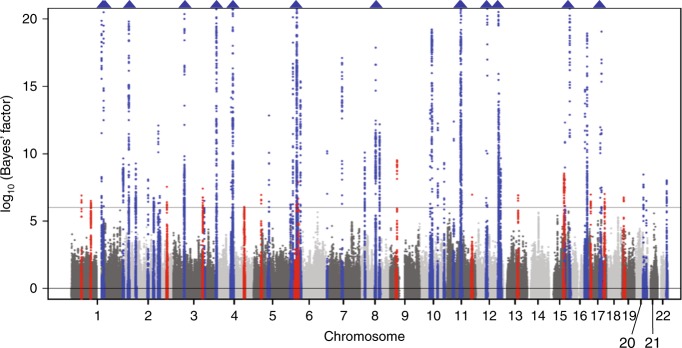
Manhattan plot for the trans-ethnic meta-analysis of SUA. The horizontal line represents the genome-wide significance level (log_10_ (Bayes’ factor) = 6). Loci shown in blue were identified by our Japanese meta-analysis and other studies and those in red indicate 15 novel loci identified in the trans-ethnic meta-analysis. Blue triangles represent loci containing SNPs with log_10_ (Bayes’ factor) of >20. SUA serum uric acid

We performed the trans-ethnic meta-analysis across our meta-analysis and the GUGC-based study to carry out fine-mapping analysis and identify further novel loci associated with SUA. We observed genome-wide significant (log_10_ (Bayes’ factor) of >6) association signals at 59 loci (Fig. 3), of which 15 were novel. Shown in Supplementary Data 7 are sentinel SNPs with the highest log_10_ (Bayes’ factor) for SUA at each of these 15 novel loci (rs302684 of *TRABD2B*-*SKINT1L*, rs2765545 of *CCDC18*, rs715 of *CPS1*, rs9942075 of *TFDP2*, rs10471103 of *INPP4B*-*LOC105377623*, rs461660 of *RAI14*, rs2760181 of *KIAA0319*, rs6928482 of *HLA-DQB1*, rs10971419 of *B4GALT1*, rs2195525 of *USP2*, rs626277 of *DACH1*, rs2957742 of *MYO9A*, rs12451900 of *ZBTB4*, rs164009 of *QRICH2*, and rs1035941 of *INSR*).

## Discussion

In a genome-wide meta-analysis performed with 121,745 Japanese subjects, we have here identified eight novel loci significantly associated with SUA. Moreover, five of these loci were replicated in Caucasian populations.

Gout, which develops as a consequence of hyperuricemia, is a form of arthritis known from the time of ancient Egypt^37^, and modern Japanese are genetically known to be more susceptible to hyperuricemia and gout,^10,38^. To our knowledge, the present study is the largest genome-wide meta-analysis performed for SUA to date, and it thus provides important insight into the genetic background of hyperuricemia and gout.

Uric acid or urate is an end metabolite of purines such as adenosine derived from ATP and guanine derived from DNA. Urate is produced predominantly in the liver and is excreted by the kidneys and the intestine^9,39,40^. Genes for urate transporters and proteins associated with cell metabolism might therefore be expected to be associated with SUA. Indeed, urate transporter genes such as *SLC22A12* (also known as *URAT1*), *SLC2A9* (*GLUT9*), and *ABCG2* (*BCRP*) have been markedly associated with SUA, hyperuricemia, and gout^7–12^.

Among the eight novel loci identified in our study, *TMEM18*, *TM4SF4*, *MXD3*, and *HNF4A* are related to cell metabolism or proliferation. *TMEM18* is a highly conserved gene related to obesity and plays a role in the central control of appetite and body weight regulation^41–43^. *TM4SF4* is associated with gallstone disease and has been implicated in both liver regeneration and pancreas development^44,45^. Both *MXD3* and *HNF4A* encode transcription factors. MXD3 forms a heterodimer with the cofactor MAX and is thought to promote uncontrolled cell proliferation and tumorigenesis^46,47^. *HNF4A* is associated with nonalcoholic steatohepatitis^48^ and plays a role in hepatic gluconeogenesis and lipid metabolism^49^. In addition, HNF4A controls gene expression in pancreatic islets, with *HNF4A* mutations having been associated with maturity-onset diabetes of the young type 1 and hyperinsulinemic hypoglycemia^50^. Furthermore, three of the eight novel loci identified in the present study harbor genes related to oxidative stress and inflammation: *SESN2*, *PSORS1C1*, and *PNPLA3*. *SESN2* encodes a highly conserved stress-inducible metabolic protein that protects cells from stressors such as hypoxia, starvation, DNA damage, and oxidative stress^51,52^. *PSORS1C1* and *PSORS1C2* encode psoriasis susceptibility 1 candidates 1 and 2, respectively. *PSORS1C1* is implicated in synovial inflammation and bone destruction in rheumatoid arthritis^53^, which, like gout, is a common type of arthritis. Its expression is inhibited in synovial fibroblasts affected by rheumatoid arthritis, which results in a reduction in interleukin-17, osteoclastogenic factor, and interleukin-1 levels as well as attenuation of cell proliferation^54^. *PNPLA3* encodes a membrane protein located at the surface of hepatocyte lipid droplets^55^. A GWAS of nonalcoholic fatty liver disease identified *PNPLA3* as a major genetic determinant of fatty liver and hepatic fat content^56^. *PNPLA3* is also associated with inflammation, fibrosis, and the development of hepatocellular carcinoma^55,57^. Thus, novel loci associated with SUA were also related to oxidative stress and inflammation. Given that uric acid has an antioxidative effect^1,2^, loci related to oxidative stress or inflammation might also be expected to be associated with SUA. However, further molecular functional analyses are required to confirm these associations. The functional relation of the last of the eight novel loci identified in the present study, *LINC01578*, to SUA is unknown. Indeed, *LINC01578* encodes a long intergenic non-protein-coding RNA of unknown function. It is also possible that a gene located near *LINC01578* is actually responsible for the observed association with SUA.

Previous candidate analyses^7,8^ and GWASs^11,12,58^ of clinically defined gout identified nonsynonymous variants of gout susceptibility genes such as *ABCG2* (rs72552713, Q126X; rs2231142, Q141K) and *GCKR* (rs1260326, L446P). *SLC22A12* (*URAT1*) and *SLC2A9* (*GLUT9*) are also genetic loci that influence SUA and encode urate transporters that mediate physiological urate reabsorption in the kidney^59,60^. We previously showed that dysfunctional nonsynonymous variants of *SLC22A12* and *SLC2A9* are responsible for renal hypouricemia type 1^59,60^ and type 2^43^, respectively. The present study also identified missense SNPs at two loci, rs738409 (I148M) of *PNPLA3* and rs74896528 (P87S) of *SESN2*, that are predicted to impair the function of the encoded proteins (Supplementary Table 4). The rs738409 (I148M) polymorphism of *PNPLA3* is in LD with rs2281293, which showed the most significant association with SUA at this locus in our genome-wide meta-analysis. The rs2281293 SNP of *PNPLA3* is also an eQTL for this gene (Supplementary Data 4). On the other hand, rs74896528 of *SESN2* has not been identified as an eQTL (Supplementary Data 4), and its SNP was not reported in the previous study based on GUGC data^24^ because of its low frequency in Caucasian populations (Supplementary Data 2). These results suggest that this missense (P87S) variant of *SESN2* (rs74896528) is a novel locus that is associated with SUA specifically in Japanese or Asian populations.

The 28 loci identified in the European population of the GUGC study, SNPs at 21 loci showed a nominal or genome-wide significant association with SUA in our meta-analysis (Supplementary Data 6), again with the same direction of effect size. The SNP-based heritability for Japanese was 14.0%, and was similar to the 14.6% seen in Europeans. The genetic correlation between Japanese and Europeans was not significantly <1. These results suggest the possibility that most genetic causal variants of SUA are shared across ancestries.

The present study also identified 132 correlated genes whose expression levels are associated with SUA-increasing alleles (Supplementary Data 5). UniProt term enrichment analysis showed that these correlated genes are enriched in genes related to “transport” (Supplementary Table 5). A novel locus, rs6031598 of *HNF4A*, is correlated with the expression level of *HNF4A*. Of note, a noncoding genetic variant, rs1967017 of *PDZK1*, which encodes a scaffold protein for urate transporters^61,62^, has been shown to be functionally linked to HNF4-dependent PDZK1 expression^63^.

For SNP rs9394948 of *ZNF318*, *ABCC10* (*MRP7*), an ABC transporter gene, was a positively correlated gene, and *SLC22A7* (*OAT2*), an SLC transporter gene, was a negatively correlated gene (Supplementary Data 5). *SLC22A7* encodes organic anion transporter 2 (OAT2), which mediates urate transport^64^ and is expressed in kidney and liver. Furthermore, for SNP rs11952102 of *MXD3*, *RAB24*, and *PRELID1* were positively correlated genes, and *MXD3* was a negatively correlated gene. RAB24 is localized to the endoplasmic reticulum and is thought to participate in autophagosome maturation^65^. RAB24 may influence SUA via autophagy, because there is a report on relationship between SUA and autophagy which is promoted by NLRP3 and results in phagocytosis of urate crystals by human osteoblasts^66^. *PRELID1* encodes PRELI, which forms a complex with TRIAP1 and mediates intramitochondrial transport of phosphatidic acid^67^. It is possible that PRELI may function as a urate transporter that directly affects SUA or that it indirectly influences SUA via its function as a phosphatidic acid transporter.

In trans-ethnic meta-analysis across our own meta-analysis and the GUGC study, we have here identified 15 more novel loci significantly associated with SUA. Out of these, rs2760181 of *KIAA0319* at 6p22.3 showed different direction of regression coefficients between Japanese and European studies, but showed genome-wide significant association (log_10_ Bayes’ factor >6). Future studies will therefore be necessary to validate our findings in independent cohorts.

The present genome-wide meta-analysis of SUA in Japan identified eight novel loci. Furthermore, trans-ethnic meta-analysis of SUA in the present study revealed 15 more novel loci associated with SUA. The present study also demonstrated that SUA is regulated by multiple “transport”-related genes, that is, not only urate transporter genes but also non-transporter genes such as *PDZK1* and *HNF4A*. Our findings thus provide important insight into SUA regulation and the pathogenesis of hyperuricemia and gout, and they provide a potential basis for the development of new treatments for these diseases.

## Methods

### Study subjects and genotyping

We performed a genome-wide meta-analysis based on three Japanese cohorts including those of the J-MICC Study^29,30^, KING Study^31,32^, and BBJ Project^33,34^. An overview of the characteristics of the study populations is provided in Supplementary Table 1. Information regarding study-specific genotyping, imputation, and analysis tools is provided in Supplementary Table 2. Data and sample collection for the cohorts participating in the present study were approved by the respective research ethics committees. All participants provided written informed consent.

### Details of cohorts

The Japan Multi-institutional Collaborative Cohort (J-MICC) Study was launched in 2005. Through March 2014, 92,642 Japanese participants aged 35 to 69 years had provided blood samples and lifestyle data based on a questionnaire after having given their informed consent^29,30^. The present study included 14,539 J-MICC Study participants randomly selected from the 12 targeted areas (Chiba, Shizuoka-Sakuragaoka, Shizuoka, Daiko, Okazaki, Aichi, Takashima, Kyoto, Tokushima, Fukuoka, Kagoshima, and Kyushu-KOPS (Kyushu Okinawa Population Study)). After preimputation quality control, 14,091 participants remained for the imputation process (Supplementary Table 2). SUA was measured with the uricase-peroxidase method or the uricase–3,5-dimethoxy-4-fluoroanilide (F-DAOS) method in 10,794 of the 14,091 participants. Individuals receiving treatment for hyperuricemia or gout were excluded. Finally, 10,621 participants remained for the association analysis (Supplementary Table 1). This study was approved by the ethics committees of Nagoya University Graduate School of Medicine (approval no. 939-14), Aichi Cancer Center, and all other participating institutions. All research procedures were conducted according to the Ethical Guidelines for Human Genome and Genetic Sequencing Research in Japan and the Declaration of Helsinki.

The Kita-Nagoya Genomic Epidemiology (KING) Study (ClinicalTrials.gov identifier NCT00262691) is an ongoing community-based prospective observational study of the genetic basis of cardiovascular disease and its risk factors^31,32^. It recruited 3975 Japanese subjects aged 50–80 years who underwent community-based annual health checkups between May 2005 and December 2007. A total of 2095 of the KING Study samples was included in the present study. SUA was measured with the uricase method (Mizuho Medy, Saga, Japan). Individuals under treatment for hyperuricemia or gout were excluded. The study was performed according to the guidelines of the Declaration of Helsinki; the study protocol was approved by the ethics committees of Aichi Gakuin University, Jichi Medical University, Nagoya University, and Kyushu University; and all participants provided written informed consent.

The BioBank Japan (BBJ) Project (http://biobankjp.org/english/index.html) was initiated in 2003 at the Institute of Medical Science, The University of Tokyo, and it has constructed a large-scale, multi-institutional, hospital-based biobank. The BBJ collected DNA, serum, and clinical information from ~200,000 Japanese patients with any of 47 target diseases between fiscal years 2003 and 2007^33,34^. Patients were recruited from 66 hospitals of 12 medical institutes throughout Japan (Osaka Medical Center for Cancer and Cardiovascular Diseases, Cancer Institute Hospital of Japanese Foundation for Cancer Research, Juntendo University, Tokyo Metropolitan Geriatric Hospital, Nippon Medical School, Nihon University School of Medicine, Iwate Medical University, Tokushukai Hospitals, Shiga University of Medical Science, Fukujuji Hospital, National Hospital Organization Osaka National Hospital, and Iizuka Hospital). All patients were diagnosed with one or more of the 47 target diseases by physicians at the cooperating hospitals. Clinical information, including SUA measurements, was collected through interviews and reviews of medical records with the use of a standard questionnaire. The present study included 109,029 individuals aged between 18 and 85 years with valid SUA measurements as described elsewhere^28^. Subjects receiving urate-lowering therapy (allopurinol, febuxostat, probenecid, or benzbromarone) or with renal insufficiency (estimated glomerular filtration rate of <15 ml min^–1^ 1.73 m^–2^) were excluded. We obtained written informed consent from all participants, and this study was approved by the ethics committees of RIKEN Center for Integrative Medical Sciences and the Institute of Medical Science, The University of Tokyo.

### Association analysis for SNPs and SUA

Individuals taking urate-lowering drugs were excluded from the present study. SUA was adjusted for age, sex, the top 10 principal components, and study-specific covariates in a linear regression model. We then standardized the resulting residuals. The association of the *z*-score of the residuals with SNP allele dose was tested by linear regression analysis. The effect sizes and standard errors estimated in linear regression analysis were used in the subsequent meta-analysis.

### Quality control after genotype imputation

After genotype imputation, quality control was applied to each study. SNPs with an imputation quality of *r*^2^ < 0.3 or a minor allele frequency of <0.005 were excluded. SNPs that passed quality control in both the J-MICC Study and BBJ cohorts were subjected to meta-analysis. To identify studies with inflated GWAS significance, which can result from population stratification, we computed the genomic control lambda^68^ and the intercept of LD score regression^69^. We calculated the genomic control lambda in R. A study showing a score of >1.1 for both measures was regarded as inflated. Inflation was not detected in any study included in the present meta-analysis, and so genomic control adjustment was not applied.

### Meta-analysis

The meta-analysis was performed with a total of 121,745 Japanese subjects from the three cohorts (Supplementary Table 1). The association results for each SNP across the studies were combined with METAL software^70^ by the fixed-effects inverse-variance-weighted method. Heterogeneity of effect sizes was assessed with the *I*^2^ index. The meta-analysis included 5,864,938 SNPs and the results from at least both the J-MICC Study and BBJ Project. The genome-wide significance level *α* was set to a *P* value <5 × 10^–8^.

### Replication study for novel loci with the GUGC-based study

To employ a replication study and compare our meta-analysis with publicly available results from Europeans conducted by the GUGC, we downloaded the summary statistics from their website. The EAF of the HapMap project phase 2 CEU samples for each SNP was added to the summary statistics of the GUGC because the results of the GUGC study did not include EAFs. We excluded variants with MAF < 0.01. *P*-values for the GUGC study were corrected for genomic control (lambda = 1.12 for SUA and 1.03 for gout)^24^. Genomic inflation did not occur in the GUGC study because the intercepts of the LD score regression, based on the raw *P*-values, were 1.01 for SUA and 1.09 for gout. We therefore calculated the raw *P* values from the corrected *P* values, and used the raw *P* values as a replication study for novel loci in our meta-analysis. For the replication of five novel loci, the significance level *α* was determined by dividing 0.05 by the number of loci for Bonferroni correction (*α* = 0.05/5 = 0.01).

### Functional annotations

For prioritization of associated SNPs at the novel loci, we adopted a series of bioinformatics approaches to collate functional annotation. We first used ANNOVAR^71^ to obtain an aggregate set of functional annotations—including gene location and impact of amino acid substitution based on the prediction tools SIFT and PolyPhen-2—for the sentinel SNPs and SNPs in high-LD (*r*^2^ of ≥0.8 in JPT of 1000 Genomes phase 3) with the sentinel SNPs and with a *P* value of <1 × 10^–6^ for SUA. We also examined these sentinel and high-LD SNPs for identification of eQTLs in 14 tissues considered relevant to SUA regulation using the GTEx v7 database. The significant criteria for eQTL were based on the GTEx project:^21^ variants with a nominal *P* value below the gene-level threshold were regarded as significant. The gene level threshold was determined by the permutation test in the GTEx project^21^. UniProt term enrichment analysis for the sets of positively correlated genes and negatively correlated genes was performed with DAVID and with the threshold of a false discovery rate of <0.05 as calculated by the Benjamini–Hochberg adjustment method.

### SNP-based heritability in Japanese and European samples

We estimated the SNP-based heritability of SUA for our Japanese meta-analysis and GUGC-based study^24^ with the use of LD score regression^69^. As explained in our replication study section, the EAF of the HapMap project phase 2 CEU samples for each SNP was added to the summary statistics of the GUGC because the results of the GUGC study did not include EAFs. The heritability estimates were calculated from the summary statistics of 1,447,573 SNPs, which were assessed in both studies and have MAF ≥ 1% in both studies and were not palindromic SNPs. The *P* values for the GUGC study were corrected for genomic control (lambda = 1.12)^24^. Genomic inflation did not occur in GUGC because the intercept of LD score regression based on the raw *P* values was 1.01. Thus, we used raw *P* values calculated from corrected *P* values. Furthermore, we calculated the genetic correlation between Japanese and Europeans using the same data sets. The genetic correlation was calculated with the use of Popcorn^72^.

### Trans-ethnic meta-analysis with the use of GUGC-based study

For our trans-ethnic meta-analysis across our meta-analysis and the GUGC-based study, we used MANTRA v.1 software^73^, which has been developed for trans-ethnic meta-analysis allowing heterogeneity in allelic effects. The trans-ethnic meta-analysis was calculated from the summary statistics of 1,986,983 SNPs, which were assessed in both studies and have MAF ≥ 1% in both. In our meta-analysis, the effect sizes were calculated from a linear regression analysis in which the *z*-score of residual values of SUA values after adjustment for covariates was used as a dependent variable. In the GUGC project, the effect sizes were calculated from the linear regression analysis in which the SUA value was used as a dependent variable. The scale of effect size for these studies was therefore different. Thus, before the MANTRA analysis, the effect sizes and standard errors of the GUGC study were divided by the standard deviation of SUA in the GUGC study (=1.4 mg/dl) to approximate the scale of effect sizes. A prior model of the relatedness between the studies was estimated by employing a dmatcal script in the software using the allele frequency of the analyzed SNPs. We regarded log_10_ Bayes’ factor >6 as a significant threshold in line with the previous simulation study^74^.

### Reporting Summary

Further information on experimental design is available in the Nature Research Reporting Summary linked to this article.

## Supplementary information

Supplementary Information

Reporting Summary

Description of Additional Supplementary Files

Supplementary Data 1

Supplementary Data 2

Supplementary Data 3

Supplementary Data 4

Supplementary Data 5

Supplementary Data 6

Supplementary Data 7

## Data Availability

The summary statistics of our genome-wide meta-analysis based on three Japanese cohorts is available at the National Bioscience Database Center (Research ID: hum0167.v1.meta.v1).
