# Supplementary material for: Genome-wide meta-analysis identifies multiple novel loci associated with serum uric acid levels in Japanese individuals
Source: Commun Biol. 2019 Apr 8;2:115. doi: 10.1038/s42003-019-0339-0 (PMC6453927; doi:10.1038/s42003-019-0339-0)
Supplement: Supplementary file 1 — Supplementary Information [file 42003_2019_339_MOESM1_ESM.pdf]

Supplementary Figures

Supplementary Fig. 1. Q-Q plot for the *P* values in the meta-analysis. The vertical and horizontal axes indicate observed and expected $-\log_{10}$(*P* value) for tests of association between SNPs and SUA, respectively. *P* values with < 1.0×10^{-320} were set to *P* value = 1.0×10^{-320}. SUA, serum uric acid.

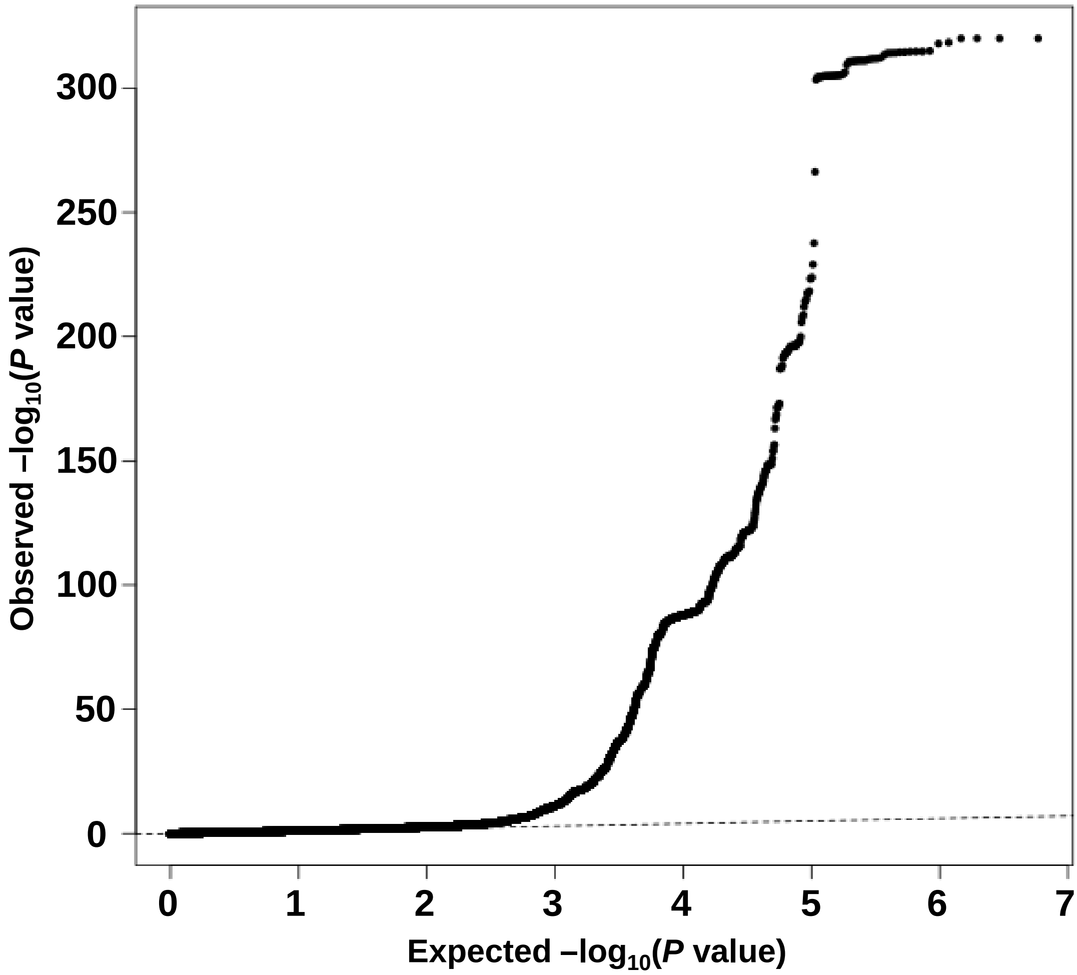

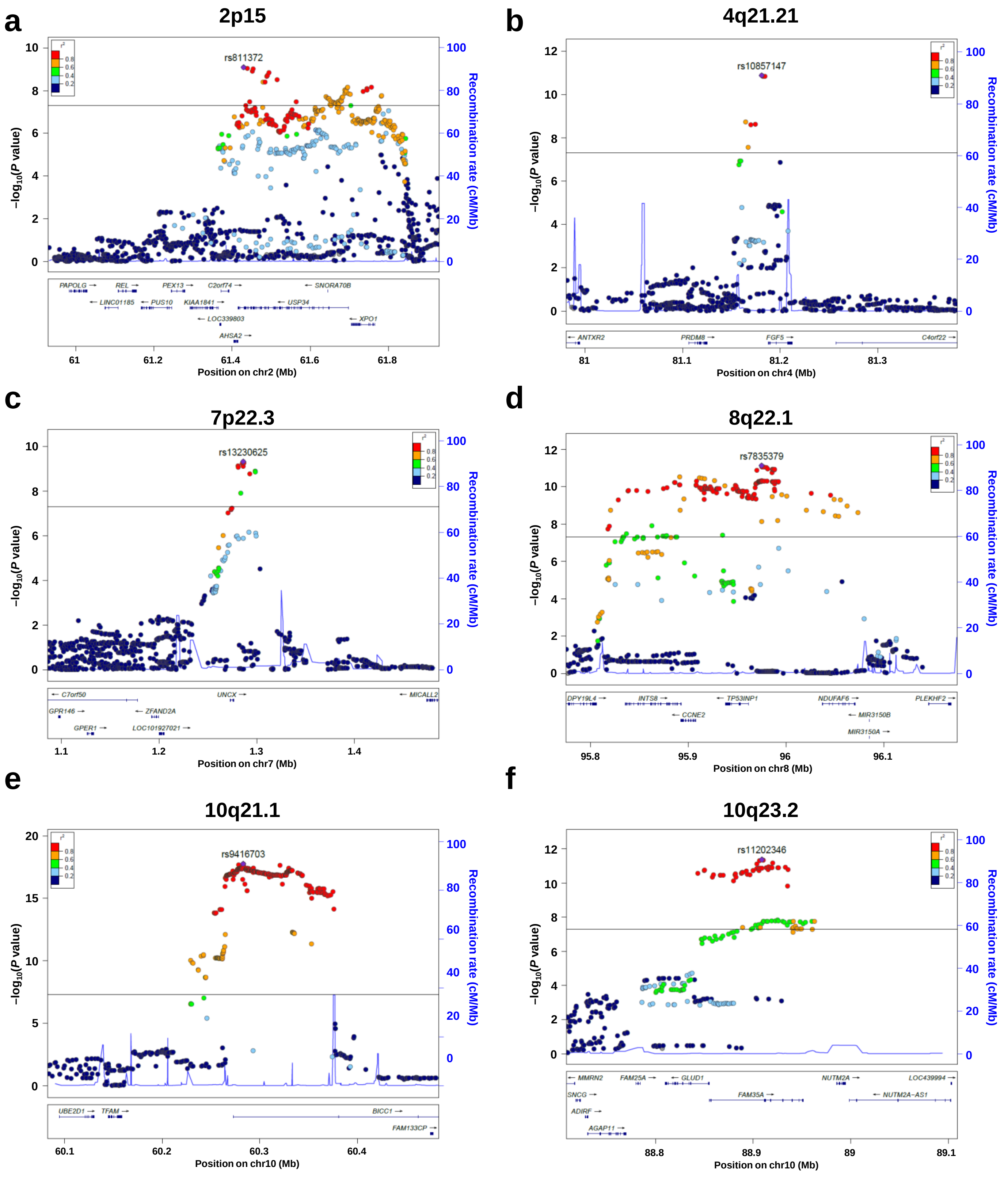

Supplementary Fig. 2. Regional association plots for the 10 loci identified in both the present and BBJ studies. Details of the 10 loci are also shown in Table 1. The vertical axis represents $-\log_{10}(P \text{ value})$ for assessment of the association of each SNP with SUA. Panels **a** to **j** present plots for 2p15, 4q21.21, 7p22.3, 8q22.1, 10q21.1, 10q23.2, 10q26.11, 11p15.4, 11p14.1, and 20q13.32, respectively. Colors indicate LD (r^2) between each sentinel SNP and neighboring SNPs based on JPT of 1000 Genomes phase 3. SUA, serum uric acid.

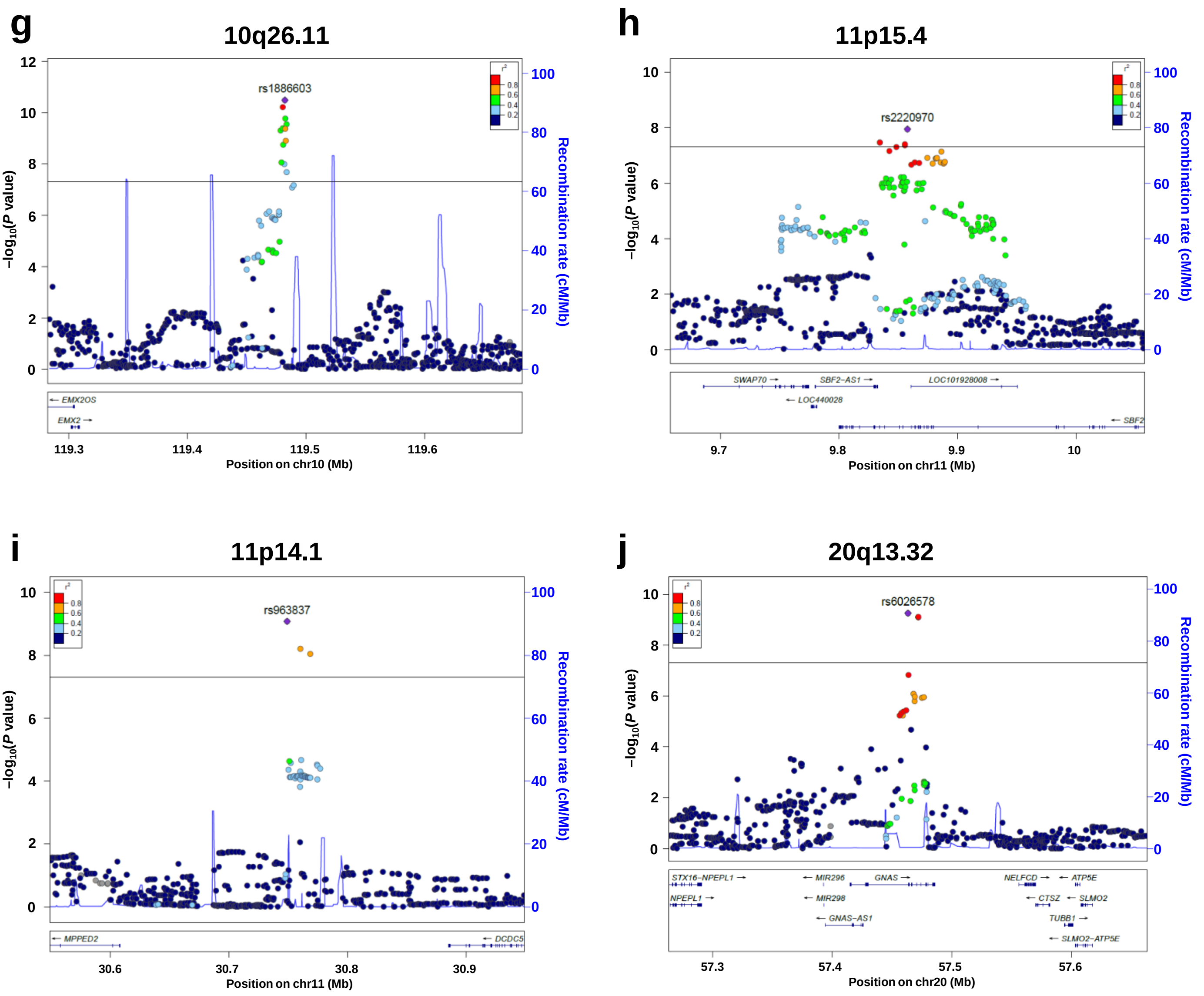

Supplementary Fig. 2 (continued)

Supplementary Table 1. Characteristics of participants

Study name	Design	Sample size[a]	Men, %	Age, Mean (SD)	BMI, Mean (SD) [kg/m^2]	SUA, Mean (SD) [mg/dl]
J-MICC	Population	10,621	44.6	54.9 (9.3)	23.0 (3.3)	5.1 (1.4)
KING-Quad	Population	475	77.7	69.5 (3.8)	23.2 (2.7)	5.6 (1.3)
KING-Omni2	Population	440	59.8	63.1 (5.3)	22.9 (2.8)	5.5 (1.4)
KING-OmniE1	Population	767	37.2	62.9 (6.3)	22.6 (2.9)	5.2 (1.3)
KING-OmniE2	Population	413	27.4	62.5 (6.4)	22.5 (3.0)	5.0 (1.2)
BBJ	Case-cohort	109,029	53.8	62.9 (13.0)	23.4 (3.7)	5.3 (1.4)

[a]Sample size indicates the number of samples that passed quality control and were subjected to the genome-wide meta-analysis of serum uric acid.

Supplementary Table 2. Genotyping, imputation, and association testing

Study name	Genotyping platform	Genotype calling algorithm	Pre-imputation QC: Sample call rate cut-off	Pre-imputation QC: other criteria	Pre-imputation QC: SNP call rate cut-off	Pre-imputation QC: SNP MAF cut-off	Pre-imputation QC: SNP HWE P cut-off	Number of Samples for imputation	Number of SNPs for imputation	Imputation reference	Imputation software	Post-imputation QC: SNP r^2 cut-off	Post-imputation QC: SNP MAF cut-off	Number of SNPs for association analysis	Association study software	Study specific covariates for association study	Genomic control lambda (LD score regression intercept)
J-MICC	Illumina HumanOmniExpressExome-8	GenomeStudio	0.99	Sex mismatches, related samples (IBD 0.1875), samples not mapping to JPT (1000 genomes)	0.98	0.01	1.00E-06	14,091	570,162	1000G phase3v5; individuals of all ancestry	SHAPEIT2 /minimac3	0.3	0.005	9,452,652	EPACTS	-	1.027 (0.995)
KING-Quad	Illumina 660W-Quad	BeadStudio	0.95	Sex mismatches, related samples (IBD 0.1875), samples not mapping to JPT (1000 genomes)	0.98	0.01	1.00E-06	494	461,200	1000G phase3v5; individuals of all ancestry	SHAPEIT2 /minimac3	0.3	0.005	9,221,314	EPACTS	−	0.993 (1.002)
KING-Omni 2	Illumina Omni2.5-8	GenomeStudio	0.95	Sex mismatches, related samples (IBD 0.1875), samples not mapping to JPT (1000 genomes)	0.98	0.01	1.00E-06	527	1,189,396	1000G phase3v5; individuals of all ancestry	SHAPEIT2 /minimac3	0.3	0.005	9,515,347	EPACTS	−	1.000 (0.986)
KING-Omni E1	Illumina OmniExpress-12	GenomeStudio	0.95	Sex mismatches, related samples (IBD 0.1875), samples not mapping to JPT (1000 genomes), duplicate samples across other KING data	0.98	0.01	1.00E-06	1,008	565,352	1000G phase3v5; individuals of all ancestry	SHAPEIT2/ minimac3	0.3	0.005	9,370,500	EPACTS	−	1.001 (1.005)
KING-Omni E2	Illumina OmniExpress-24	GenomeStudio	0.95	Sex mismatches, related samples (IBD 0.1875), samples not mapping to JPT (1000 genomes)	0.98	0.01	1.00E-06	615	533,819	1000G phase3v5; individuals of all ancestry	SHAPEIT2/ minimac3	0.3	0.005	9,321,796	EPACTS	−	0.987 (0.996)
BBJ	Illumina HumanOmniExpressExome or IlluminaHumanOmniExpress + IlluminaHumanExome	GenomeStudio	0.98	Sex mismatches, related samples (estimated by IBS), PCA outliers not clustered with East Asians (HapMap II: JPT + CHB)	0.99	0.01	1.00E-06	200,849	508,642	1000G phase1v3; individuals of East Asian ancestry	MACH/ minimac	0.7	0.005	5,925,843	mach2qtl	47 affection status	1.157 (1.033)

Supplementary Table 3. Replication analysis performed with the data of the GUGC-based GWAS for SNPs identified by the current meta-analysis of SUA

Locus	Sentinel SNP	r^{2a}	SNP	Chr	Position	Gene	Alleles		Our meta-analysis (Japanese)					GUGC-based GWAS (European)									
														Uric acid					Gout				
							Effect	Noneffect	EAF	Beta[b]	SE	*P* value	I^2	N	EAF[d]	Beta[c]	SE	*P* value[e]	N	EAF[d]	*OR*[f]	(95%CI)	*P* value[e]
1p35.3	no matched SNPs																						
2p25.3	rs10188118	1.000	rs10188118	2	653623	*LOC105373352, TMEM18*	C	G	0.864	0.035	0.006	8.60E-09	48.7	109084	0.700	0.015	0.006	1.08E-02	69374	0.700	1.05	(0.98, 1.13)	1.88E-01
3q25.1	rs6774054	1.000	rs6774054	3	149211699	*TM4SF4*	A	G	0.337	0.024	0.004	1.58E-08	0	110041	0.083	0.015	0.012	1.94E-01	69374	0.083	1.19	(1.04, 1.35)	1.18E-02
5q35.3	rs11952102	1.000	rs6885410	5	176740996	*MXD3, LMAN2*	A	C	0.447	0.022	0.004	5.20E-08	0	110148	0.750	0.015	0.006	9.73E-03	69304	0.750	1.00	(0.93, 1.07)	9.76E-01
6p21.33	no matched SNPs																						
15q26.1	no matched SNPs																						
20q13.12	rs6031598	1.000	rs6031598	20	43056149	*HNF4A*	T	G	0.378	-0.023	0.004	2.90E-08	27.5	101293	0.559	-0.018	0.006	1.73E-03	69374	0.559	0.96	(0.90, 1.03)	2.62E-01
22q13.31	rs2281293	0.984	rs12483959	22	44325996	*PNPLA3*	A	G	0.450	-0.023	0.004	7.53E-09	0	108262	0.167	-0.019	0.007	7.34E-03	69348	0.167	0.92	(0.84, 1.00)	5.59E-02

[a]The r^2 value represents the linkage disequilibrium correlation coefficient with the sentinel SNP in JPT of 1000 Genomes phase 3.

[b]The beta value represents change in z-score per effect allele copy for the SNP.

[c]The beta value represents change in serum uric acid (SUA) per effect allele copy for the SNP.

[d]EAF for GUGC was calculated from HapMap phase II r24 CEU samples.

[e]*P* values were calculated back from *P* values corrected for genomic control (lambda=1.12 for SUA and 1.03 for gout).

[f]The odds ratio (OR) value represents increased risk of gout per effect allele copy for the SNP.

Supplementary Table 4. Nonsynonymous variants at the newly identified loci of SUA

Locus	Sentinel SNP	r^{2a}	SNP	Chr	Position	Alleles		Our meta-analysis					Amino acid change						
						Effect	Non-effect	EAF	Beta[b]	SE	*P* value	I^2	Gene	Feature	Change in DNA sequence	Amino acid change	SIFT	PolyPhen2 HVAR	PolyPhen2 HDIV
1p35.3	rs74896528	1.000	rs74896528	1	28598287	T	C	0.057	-0.057	0.010	8.42E-09	0	*SESN2*	NM_031459.4	C259T	P87S	Damaging (0.0400)	Probably damaging (0.999)	Probably damaging (1.000)
22q13.31	rs2281293	0.952	rs738409	22	44324727	C	G	0.546	0.023	0.004	8.15E-09	0	*PNPLA3*	NM_025225.2	C444G	I148M	Damaging (0.0300)	Probably damaging (0.944)	Probably damaging (0.994)

[a]The r^2 value represents the linkage disequilibrium correlation coefficient with the sentinel SNP in JPT of 1000 Genomes phase 3.

[b]The beta value represents change in z-score per effect allele copy for the SNP.

SUA, serum uric acid.

Supplementary Table 5. UniProt term enrichment analysis with DAVID

Term	Count	*P* value	FDR	Genes
**Positively correlated genes**				
Williams-Beuren syndrome	4	8.15E-05	0.012	*TBL2, BAZ1B, GTF2IRD1, BCL7B*
Sodium	5	4.72E-04	0.018	*SLC17A3, SLC17A4, SLC17A1, SLC5A6, HCN3*
Transport	16	4.28E-04	0.021	*XPO1, SLC2A9, RFT1, SNUPN, SNX17, ABCC10, LMAN2, PRELID1, SLC17A3, SLC17A4, SLC17A1, PKD2, RAB24, SLC5A6, POM121B, HCN3*
Sodium transport	5	3.78E-04	0.028	*SLC17A3, SLC17A4, SLC17A1, SLC5A6, HCN3*
Alternative splicing	44	1.12E-03	0.034	*GPN1, FGFR4, MICB, NDUFAF6, MICA, EFNA1, RUSC1, SNX17, NT5DC2, CCNE2, CCHCR1, INTS8, WWP2, PKD2, USP34, GBA, BCL7B, GNL3, MUC1, SLC2A9, CRIP3, ABCC10, PNPLA3, SFMBT1, RGS14, PRELID1, MAN2C1, YY1AP1, DOK3, BAZ1B, SLC17A3, CLEC18C, SLC17A4, GTF2IRD1, SLC17A1, PKLR, COMMD2, RIT1, WDR1, NEK4, PDZK1, PHF7, ADAM15, TP53INP1*
**Negatively correlated genes**				
Williams-Beuren syndrome	4	9.00E-05	0.012	*MLXIPL, VPS37D, NSUN5P2, BCL7B*

False discovery rate (FDR) was calculated using the Benjamini-Hochberg adjustment method.
