# Supplementary material for: Genome-wide meta-analysis identifies multiple novel loci associated with serum uric acid levels in Japanese individuals
Source: Commun Biol. 2019 Apr 8;2:115. doi: 10.1038/s42003-019-0339-0 (PMC6453927; doi:10.1038/s42003-019-0339-0)
Supplement: Supplementary file 2 — Reporting Summary [file 42003_2019_339_MOESM2_ESM.pdf]

nature research

Corresponding author(s): Hirotaka Matsuo

Last updated by author(s): Dec 6, 2018

Reporting Summary

Nature Research wishes to improve the reproducibility of the work that we publish. This form provides structure for consistency and transparency in reporting. For further information on Nature Research policies, see Authors & Referees and the Editorial Policy Checklist.

Statistics

For all statistical analyses, confirm that the following items are present in the figure legend, table legend, main text, or Methods section.

n/a	Confirmed	
☐	☒	The exact sample size (*n*) for each experimental group/condition, given as a discrete number and unit of measurement
☐	☒	A statement on whether measurements were taken from distinct samples or whether the same sample was measured repeatedly
☐	☒	The statistical test(s) used AND whether they are one- or two-sided<br>*Only common tests should be described solely by name; describe more complex techniques in the Methods section.*
☐	☒	A description of all covariates tested
☐	☒	A description of any assumptions or corrections, such as tests of normality and adjustment for multiple comparisons
☐	☒	A full description of the statistical parameters including central tendency (e.g. means) or other basic estimates (e.g. regression coefficient) AND variation (e.g. standard deviation) or associated estimates of uncertainty (e.g. confidence intervals)
☐	☒	For null hypothesis testing, the test statistic (e.g. *F*, *t*, *r*) with confidence intervals, effect sizes, degrees of freedom and *P* value noted<br>*Give P values as exact values whenever suitable.*
☐	☒	For Bayesian analysis, information on the choice of priors and Markov chain Monte Carlo settings
☒	☐	For hierarchical and complex designs, identification of the appropriate level for tests and full reporting of outcomes
☐	☒	Estimates of effect sizes (e.g. Cohen's *d*, Pearson's *r*), indicating how they were calculated

Our web collection on statistics for biologists contains articles on many of the points above.

Software and code

Policy information about availability of computer code

Data collection	BeadStudio, GenomeStudio v2011<br>Details about softwares used to collect data have been included in Supplementary Table 2.
Data analysis	SHAPEIT v1, SHAPEIT v2, minimac, minimac3, mach2qtl v113, EPACTS v3.3, METAL-20110325, LDSC-170205, R v3.5, Popcorn v0.9.9<br>Details about softwares used to analyze data have been included in the Methods section of our manuscript.

For manuscripts utilizing custom algorithms or software that are central to the research but not yet described in published literature, software must be made available to editors/reviewers. We strongly encourage code deposition in a community repository (e.g. GitHub). See the Nature Research guidelines for submitting code & software for further information.

Data

Policy information about availability of data

All manuscripts must include a data availability statement. This statement should provide the following information, where applicable:

- Accession codes, unique identifiers, or web links for publicly available datasets
- A list of figures that have associated raw data
- A description of any restrictions on data availability

We already contacted to the office of National Bioscience Database Center and confirmed that our summary statistics file will be publicly available.

Field-specific reporting

Please select the one below that is the best fit for your research. If you are not sure, read the appropriate sections before making your selection.

☒ Life sciences ☐ Behavioural & social sciences ☐ Ecological, evolutionary & environmental sciences

Life sciences study design

All studies must disclose on these points even when the disclosure is negative.

Sample size	We used existing cohort data. Thus, no sample-size calculation was performed.
Data exclusions	Individuals taking urate-lowering drugs were excluded from the present study. Individuals that didn't pass quality control criteria for the SNP array in each cohort were also excluded from the study. The QC criteria was shown in Supplementary Table 2.
Replication	To replicate our findings, we used publicly available results from Europeans conducted by the Global Urate Genetics Consortium. We downloaded the summary statistics from their website.
Randomization	Our study is an observational study. Thus, no randomization was performed.
Blinding	Our study is an observational study. Thus, no blinding was performed.

Reporting for specific materials, systems and methods

We require information from authors about some types of materials, experimental systems and methods used in many studies. Here, indicate whether each material, system or method listed is relevant to your study. If you are not sure if a list item applies to your research, read the appropriate section before selecting a response.

Materials & experimental systems

n/a	Involved in the study	
☒	☐	Antibodies
☒	☐	Eukaryotic cell lines
☒	☐	Palaeontology
☒	☐	Animals and other organisms
☐	☒	Human research participants
☐	☒	Clinical data

Methods

n/a	Involved in the study	
☒	☐	ChIP-seq
☒	☐	Flow cytometry
☒	☐	MRI-based neuroimaging

Human research participants

Policy information about studies involving human research participants

Population characteristics	In all cohorts used for our meta-analysis, Japanese subjectes were recruited. Details on the subjects used in this study have been included in the Methods section of our manuscript and Supplementary Table 1.
Recruitment	Details on the subjects used in this study have been included in the Methods section of our manuscript and Supplementary Table 1.
Ethics oversight	Details about the ethics oversight have been included in the Supplementary Methods.

Note that full information on the approval of the study protocol must also be provided in the manuscript.

Clinical data

Policy information about clinical studies

All manuscripts should comply with the ICMJE guidelines for publication of clinical research and a completed CONSORT checklist must be included with all submissions.

Clinical trial registration	*Provide the trial registration number from ClinicalTrials.gov or an equivalent agency.*
Study protocol	*Note where the full trial protocol can be accessed OR if not available, explain why.*
Data collection	*Describe the settings and locales of data collection, noting the time periods of recruitment and data collection.*
Outcomes	*Describe how you pre-defined primary and secondary outcome measures and how you assessed these measures.*
