# Supplementary material for: Genome-wide meta-analysis identifies multiple novel loci associated with serum uric acid levels in Japanese individuals
Source: Commun Biol. 2019 Apr 8;2:115. doi: 10.1038/s42003-019-0339-0 (PMC6453927; doi:10.1038/s42003-019-0339-0)
Supplement: Supplementary file 3 — Description of Additional Supplementary Files [file 42003_2019_339_MOESM3_ESM.docx]

**Description of Additional Supplementary Files**

**File Name**: Supplementary Data 1

**Description**: Sentinel SNPs associated with SUA in each of Japanese studies as identified in the meta-analysis.

**File Name**: Supplementary Data 2

**Description**: Effect allele frequencies of sentinel SNPs associated with SUA in Japanese for 1000 Genomes phase 3.

**File Name**: Supplementary Data 3

**Description**: Comparison of results obtained for 27 SNPs identified in the BBJ study between this previous study and the current meta-analysis of SUA

**File Name**: Supplementary Data 4

**Description**: Expression QTLs associated with the sentinel SNPs at the novel identified loci of SUA

**File Name**: Supplementary Data 5

**Description**: Genes whose expression level is associated with SUA-associated SNPs

**File Name**: Supplementary Data 6

**Description**: Results in the present meta-analysis for SNPs identified in the GUGC-based GWAS of SUA in subjects of European ancestry

**File Name**: Supplementary Data 7

**Description**: Sentinel SNPs associated with SUA as identified in the trans-ethnic meta-analysis
